# A network meta-analysis of 12,116 individuals from randomized controlled trials in the treatment of depression after acute coronary syndrome

**DOI:** 10.1371/journal.pone.0278326

**Published:** 2022-11-30

**Authors:** Grace En Hui Lim, Ansel Tang, Yip Han Chin, Jie Ning Yong, Darren Tan, Phoebe Tay, Yu Yi Chan, Denzel Ming Wei Lim, Jun Wei Yeo, Kai En Chan, Kamala Devi, Colin Eng Choon Ong, Roger S. Y. Foo, Huay-Cheem Tan, Mark Y. Chan, Roger Ho, Poay Huan Loh, Nicholas W. S. Chew

**Affiliations:** 1 Lee Kong Chian School of Medicine, Nanyang Technological University, Singapore, Singapore; 2 Yong Loo Lin School of Medicine, National University of Singapore, Singapore, Singapore; 3 Alice Lee Centre for Nursing Studies, Yong Loo Lin School of Medicine, National University of Singapore, Singapore, Singapore; 4 Department of Emergency Medicine, Ng Teng Fong General Hospital, Singapore, Singapore; 5 Department of Cardiology, National University Heart Centre, National University Hospital, Singapore, Singapore; 6 Department of Psychological Medicine, Yong Loo Lin School of Medicine, National University of Singapore, Singapore, Singapore; 7 Institute of Health Innovation and Technology (iHealthtech), National University of Singapore, Singapore, Singapore; Prince Sattam Bin Abdulaziz University, College of Applied Medical Sciences, SAUDI ARABIA

## Abstract

**Background:**

Post-acute coronary syndrome (ACS) depression is a common but not well understood complication experienced by ACS patients. Research on the effectiveness of various therapies remains limited. Hence, we sought to conduct a network meta-analysis to assess the efficacy of different interventions for post-ACS depression in improving patient outcomes.

**Methods and findings:**

Three electronic databases were searched for randomised controlled trials describing different depression treatment modalities in post-ACS patients. Each article was screened based on inclusion criteria and relevant data were extracted. A bivariate analysis and a network meta-analysis was performed using risk ratios (RR) and standardized mean differences (SMD) for binary and continuous outcomes, respectively.

A total of 30 articles were included in our analysis. Compared to standard care, psychosocial therapy was associated with the greatest reduction in depression scores (SMD:-1.21, 95% CI: -1.81 to -0.61, p<0.001), followed by cognitive behavioural therapy (CBT) (SMD: -0.75, 95% CI: -0.99 to -0.52, p<0.001), antidepressants (SMD: -0.73, 95% CI: -1.14 to -0.31, p<0.001), and lastly, combination therapy (SMD: -0.15, 95% CI: -0.28 to -0.03, p = 0.016). No treatment modalities was found to be more effective in reducing depression scores when compared to one another. Additional analysis showed that these treatment modalities did not have significant impact on the overall mortality, cardiac mortality and recurrent myocardial infarction.

**Conclusion:**

This network meta-analysis found that the treatment effect of the various psychological modalities on depression severity were similar. Future trials on psychological interventions assessing clinical outcomes and improvement in adherence to ACS-specific interventions are needed.

## Introduction

Acute coronary syndrome (ACS) remains one of the leading causes of mortality and morbidity in the world [1]. While there has been significant progress in the optimization of medical treatment after ACS, the psychological wellbeing of patients are often forgotten with an estimated 46.7% of the patients affected by depression after an ACS event [2–4]. While the exact mechanisms linking depression and ACS complications are not well understood [5], studies consistently show that the development of depression after ACS can be detrimental to patients both physically and psychologically [6,7]. It is estimated that ACS patients with depression are subjected to a two-fold increase in mortality risk and 1.6-fold increase in risk of adverse cardiovascular events, along with higher rate of hospitalization [6,8,9]. Furthermore, post-ACS depression are associated with medical noncompliance and a sedentary lifestyle, both of which are risk factors for recurrent cardiac events [10,11].

Currently, guidelines from the American Heart Association (AHA) and American Academy of Family Physicians (AAFP) recommend the use of antidepressants and cognitive behavior therapy (CBT) for the treatment of post-ACS depression [12,13]. Meta-analyses by Ha et al and Fernandes et al found that selective serotonin reuptake inhibitors (SSRIs) improve depression scores and can reduce the risk of some cardiac complications associated with post-ACS depression [14,15]. CBT, a form of psychotherapy involving dedicated counselling sessions aimed at assessing and addressing the cognitive and behavioral aspects of the patient, thereby enabling patients to recognize and challenge their maladaptive thought processes, was found to be an efficacious treatment option for depression after ACS [12,16]. Psychosocial therapy constituted of various methods including group counselling, education and lifestyle change programmes which generally provide flexibility in the management of the patient where the care can be tailored according to patient’s changing needs [17]. In addition, there exists a multitude of other interventions for post-ACS depression all with varying effectiveness. Examples include supplements such as omega-3 fatty acids, n-3 polyunsaturated fatty acids, and probiotics [18–20], and teleinterventions [21]. However, while these therapies have been shown to be effective measures for post-ACS depression, their comparative effectiveness is less well-understood due to a lack of direct head-to-head comparison studies [12]. Additionally, there is limited research on the relationship between therapy for post-ACS depression and hard clinical endpoints. Hence, this network meta-analysis sought to assess the efficacy of the interventions for post-ACS depression in improving depression scores, as well as mortality and cardiovascular outcomes.

## Materials and methods

### Search strategy

In accordance to the Preferred Reporting Items for Systematic Reviews and Meta-Analyses (PRISMA) guidelines [22], three major electronic databases (Medline, Embase and PsycINFO) were searched for randomised controlled trials relating to treatments for depression in post-ACS patients on 26 July 2021. A copy of the search strategy can be found in S1 Table. To ensure a comprehensive search of literature, references of related reviews and included articles were screened for relevant articles. Following the initial search, duplicates were removed using Endnote X9 prior to screening and article selection.

### Selection criteria and data extraction

Four authors (GEHL, AT, YHC and ASM) independently screened all articles and did the full text review according to the selection criteria, with a fifth independent author (NWSC) resolving any discrepancies through a consensus. Only original research articles were included, with study protocols, reviews, letters, and conference abstracts being excluded. All included studies were randomized controlled trials (RCTs), and retrospective and prospective cohorts were excluded. To generalize the findings, intervention groups were classified with clinical consultation from a psychiatrist (RH). There were 7 groups of interventions included, namely: (1) standard care; (2) psychosocial therapy; (3) antidepressants; (4) supplements; (5) CBT; (6) tele-intervention; (7) combination therapy, a combination of antidepressants with CBT. Standard care was defined as patients in the control arm of studies without intervention, while ACS as conditions resulting in reduced blood flow to the heart, inclusive of unstable angina and myocardial infarction (MI) [23]. Psychosocial therapy was defined as therapy designed to assist patients’ re-integration into society through counselling, education, or a change in lifestyle [24]. CBT was focused on correcting the maladaptive thought processes affecting the patient’s behaviour and functionality [25,26]. Antidepressants were inclusive of any pharmacological drugs administered to treat depression [27]. Supplements consisted of non-pharmacological agents that complemented the diet of patients including omega-3 fatty acids, n-3 polyunsaturated fatty acids, and probiotics [18–20]. Tele-intervention entails the use of telephone-based care aimed at improving patients’ mental state through health coaching and cognitive restructuring [21]. The four authors working in pairs extracted data that included but not limited to author, year, country, treatment duration, sample size, patients’ age, gender and mean depression scores and the depression scale used. When mean and standard deviation was not provided, an estimation was calculated using a formula provided by Wan et al. [28] The primary outcome of interest was reduction in depression scores. The secondary outcomes included overall mortality, cardiac mortality, and myocardial infarction (MI).

### Risk of bias assessment

Quality assessment was conducted using Cochrane Risk-of-Bias 2.0 (RoB2) for RCTs. RoB2 analyses the risk of bias by grading the quality of evidence through 5 bias domains including randomization process, deviation from intended outcomes, missing outcome data, outcome measurement and reported result selection [29].

### Statistical analysis

The primary outcome measure was standard mean difference (SMD) that accounts for variability in the type of instrument measures used to objectify depression. Binary outcomes were analysed in risk ratio (RR). Firstly, a standard bivariate analysis was conducted in RStudio (Version 1.4.1717) using the *meta* package. The inverse variance method and Hedges’ g were utilized to pool the results and obtain a weighted mean effect. Mantel-Haenszel RR estimates with corresponding 95% confidence intervals (CI) were pooled using the DerSimonian and Laird model [30]. Heterogeneity measures were quantified from within-design Q statistic (of which p<0.10 indicates significant heterogeneity) and the heterogeneity statistic I^2^. The I^2^ measures the degree of heterogeneity within our analysis, with an I^2^ value of 25%, 50% and 75% equating to small, moderate and large amounts of heterogeneity respectively [31]. Next, a network meta-analysis was then performed in STATA 16.1 with reference to White et al to draw indirect comparisons between the treatment groups [32]. A network diagram was plotted, and the thickness of each line represents the number of studies included in the analysis [33]. Binary outcomes were first log-transformed prior to pooling and exponentiated to obtain the RR. To analyse the validity of the indirect comparisons, consistency was measured using local nodes splitting and Wald testing [34,35]. Publication bias was addressed through a visual inspection of the funnel plot (S1 Appendix). Quality and certainty of the individual outcomes of the meta-analysis were assessed using the Grading of Recommendations, Assessment, Development and Evaluations (GRADE) framework [36].

## Results

### Summary of included articles

**Fig 1:**
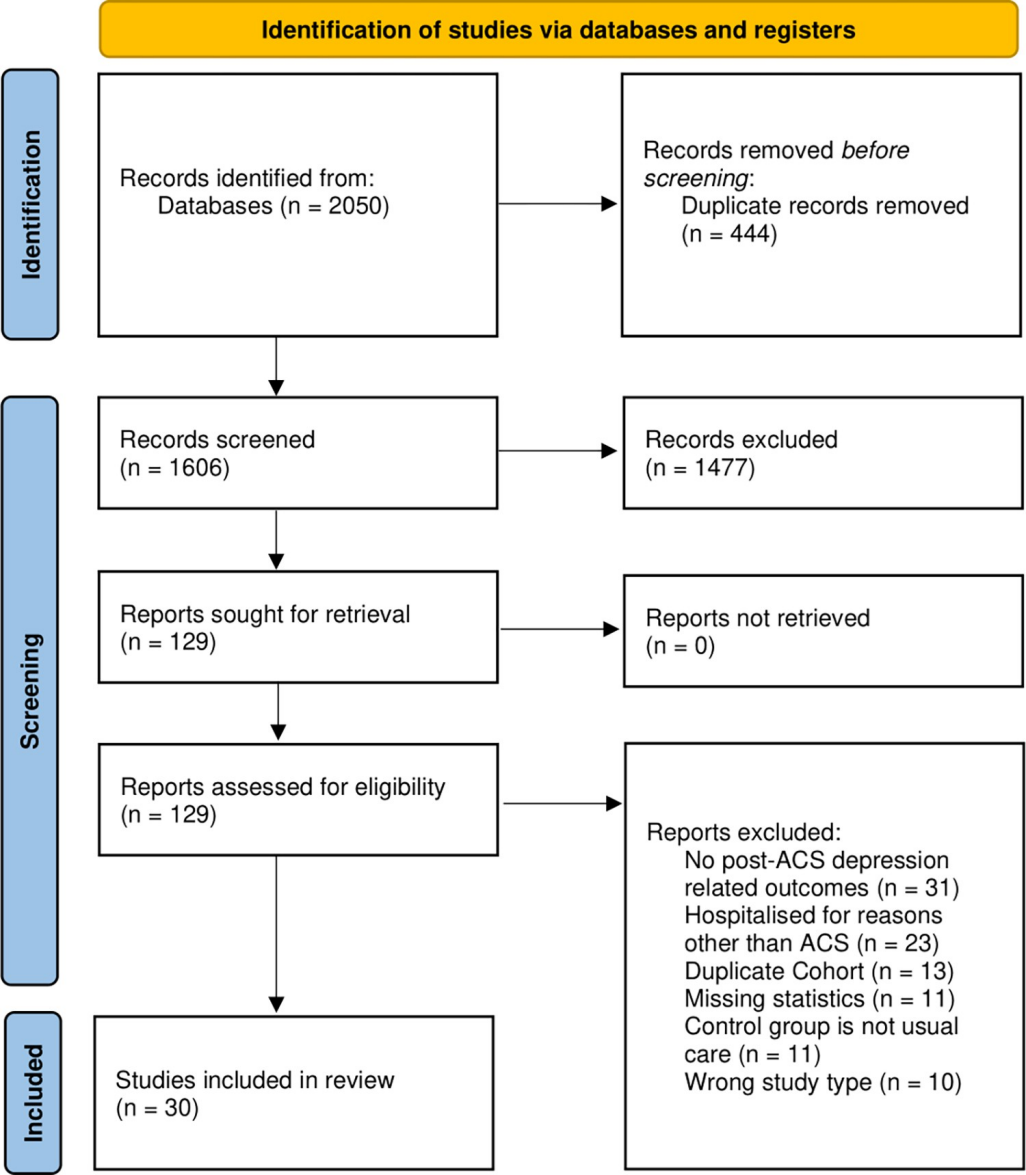
PRISMA flow diagram.

A total of 2,050 articles were identified in the initial search strategy. After removal of duplicates, 1,606 articles were screened, of which 129 articles were included for full text review. In total, 30 articles were included in this meta-analysis (Fig 1). Ten articles originated from USA [25,37–45], 3 from Australia [24,46,47], Iran [48–50] and Netherlands [51–53], 2 from Canada [27,54] and Italy [55,56], and 1 each from China [57], India [58], Poland [59], Portugal [60], Singapore [20], South Korea [61], and Sweden [62]. All 30 articles were RCTs with low to moderate risk of bias (S2 Table). In total, there were 12,116 patients included in the analysis, with the mean age ranging from 49.9 to 68.8 and proportion of female from 0.050 to 0.679. Of all patients, 3,118 received supplements [25,49,59], 1,619 received CBT [27,37–39,47,48,55,62], 1,123 patients received psychosocial therapy [20,43–45,50,52,54,56,57,60] 597 received antidepressants [40,41,51,53,58,61], and 202 received tele-intervention [24,46]. Combination therapy, a combination of antidepressants with CBT, was used in a study of 499 patients by Kronish et al. [42] The mean duration of follow-up was 10.6 months (Standard Deviation: 9.6). The summary of included articles can be found in S3 Table, and a summary of the direct to indirect comparisons for each network meta-analysis can be seen in S2 Appendix.

### Reduction in depression scores

**Fig 2:**
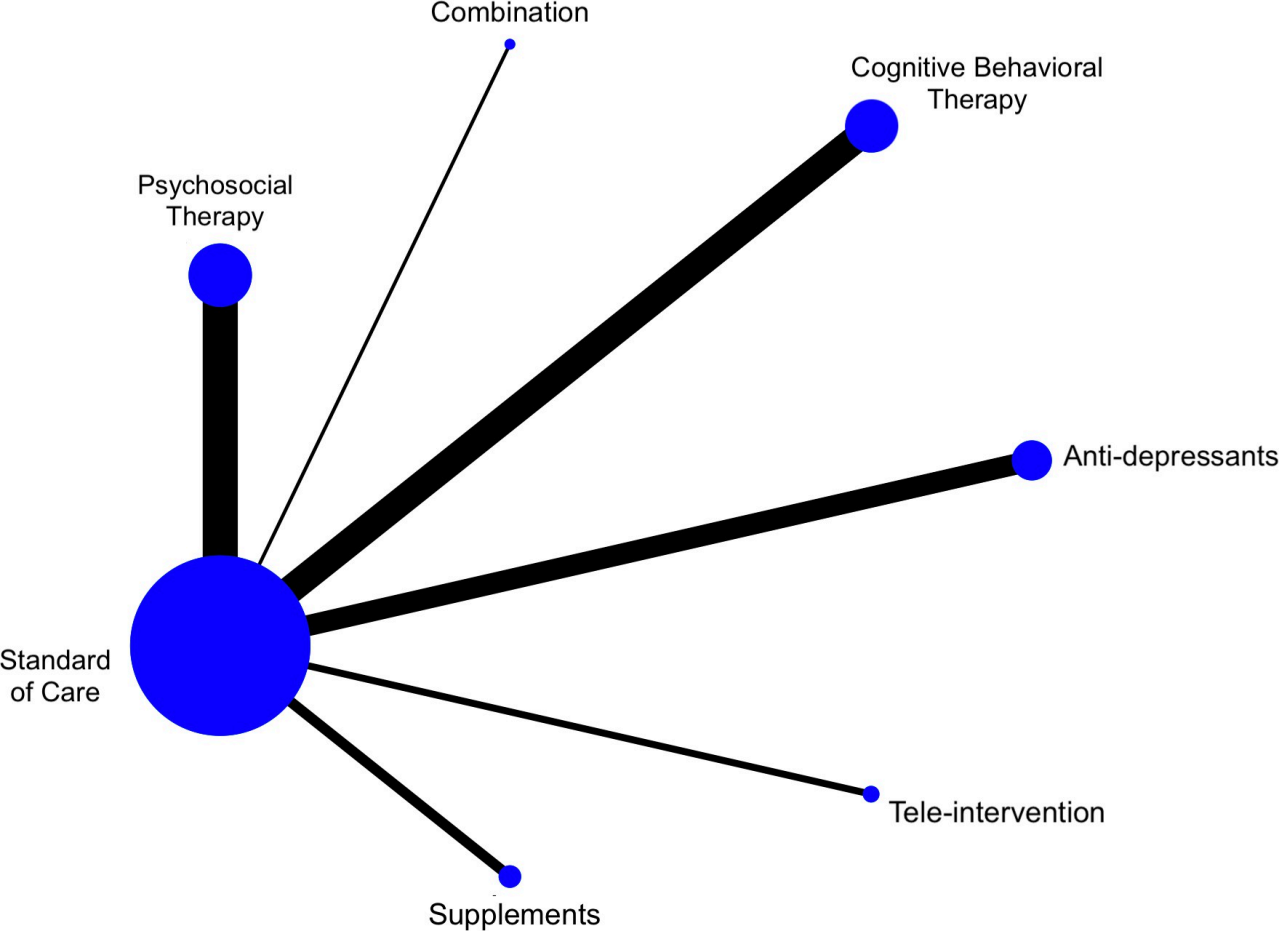
Network diagram.

**Fig 3:**
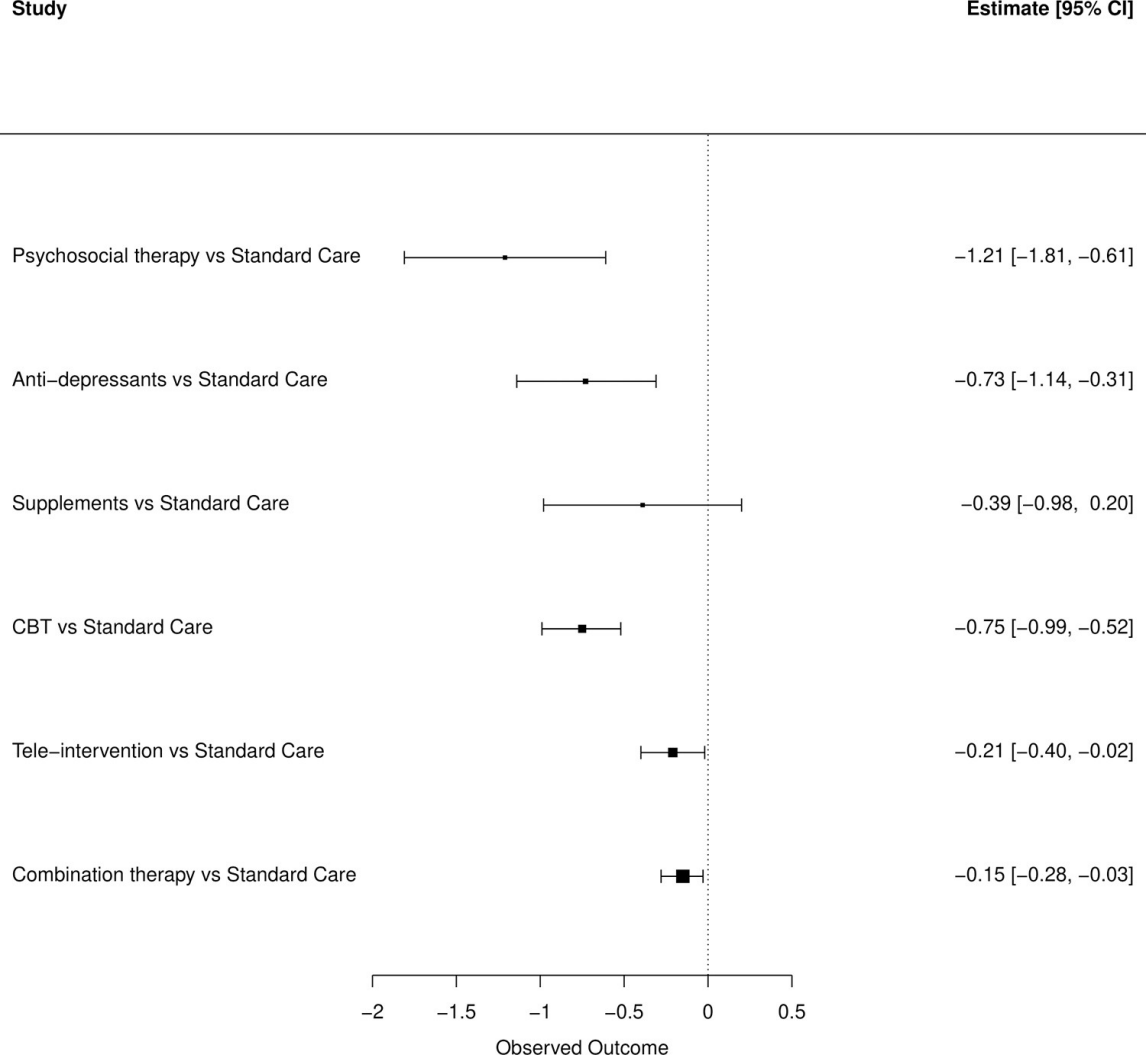
Forest plot of bivariate analysis for depression scores.

The network diagram depicts the comparison of the standard care to the different treatment modalities for the reduction in depression scores (Fig 2). Compared to standard care, the reduction of depression scores in all treatment modalities was statistical significance except for supplements, with moderate to high certainty of results following the GRADE score (Table 1, Fig 3). The largest reduction was observed with psychosocial therapy when compared to standard care (SMD: -1.21, 95% CI: -1.81 to -0.61, p<0.001) followed by CBT (SMD: -0.75, 95% CI: -0.99 to -0.52, p<0.001), antidepressants (SMD: -0.73, 95% CI: -1.14 to -0.31, p<0.001), and lastly combination therapy (SMD: -0.15, 95% CI: -0.28 to -0.03, p = 0.016). When comparing between the treatment modalities, there were no significant differences between the six treatment modalities in reducing depression, and there was high levels of heterogeneity noted (I^2^ = 97.4%) between the comparisons (Table 2). Compared to psychosocial therapy, the reduction in depression scores with antidepressants (SMD: 0.47, 95% CI: -0.67 to 1.61, p = 0.422) and CBT (SMD: 0.36, 95% CI: -0.68 to 1.41, p = 0.497) did not differ significantly. Moreover, the reduction in depression scores with antidepressants and CBT (SMD: 0.10, 95% CI: -1.08 to 1.29, p = 0.863) was also similar.

**Table 1:** Summary of bivariate analysis.

Intervention vs Standard of care	Studies	Total	I^2^	SMD/RR (95% CI)	P-value	GRADE score
**Depression scores**						
Psychosocial therapy	10	2193	96.20%	-1.21 (-1.81 to -0.61)	<0.001	High
Antidepressants	6	1042	86.20%	-0.73 (-1.14 to -0.31)	<0.001	Moderate
Supplements	3	4212	82.80%	-0.39 (-0.98 to 0.20)	0.195	High
CBT	8	3252	75.90%	-0.75 (-0.99 to -0.52)	<0.001	High
Tele-intervention	2	418	0.00%	-0.21 (-0.40 to -0.02)	0.033	Moderate
Combination therapy	1	999	-	-0.15 (-0.28 to -0.03)	0.016	Moderate
**Overall Mortality**						
Psychosocial therapy	5	1729	0.00%	1.34 (0.83 to 2.16)	0.227	High
Antidepressants	3	477	0.00%	0.54 (0.13 to 2.29)	0.406	Moderate
CBT	1	2481	-	0.98 (0.80 to 1.19)	0.847	Moderate
Combination therapy	1	999	-	1.28 (0.70 to 2.34)	0.423	Moderate
**Cardiac Death**						
Psychosocial therapy	4	1665	-	1.42 (0.84 to 2.39)	0.189	Moderate
CBT	2	2581	0.00%	0.84 (0.65 to 1.09)	0.184	Moderate
**Myocardial Infarction**						
Psychosocial therapy	2	1440	0.00%	1.00 (0.63 to 1.58)	0.998	Moderate
Antidepressants	2	386	0.00%	0.84 (0.35 to 2.01)	0.693	Moderate
CBT	1	2481	-	0.99 (0.81 to 1.21)	0.939	Moderate

P-value <0.05 is significant; *- Values given in SMD (95%CI); CBT, cognitive based therapy; SMD, standard mean difference; RR, Risk Ratio.

**Table 2:** Summary of network analysis for depression scores.

	Psychosocial therapy	Antidepressants	Supplements	CBT	Tele-intervention	Combination therapy
**Psychosocial therapy**	-	-0.47 (-1.61 to 0.67, p = 0.420)	-0.80 (-2.24 to 0.64, p = 0.276)	-0.36 (-1.41 to 0.68, p = 0.495)	-1.06 (-2.72 to 0.60, p = 0.211)	-1.08 (-3.29 to 1.13, p = 0.339)
**Antidepressants**	0.47 (-0.67 to 1.61, p = 0.422)	-	-0.33 (-1.87 to 1.21, p = 0.673)	0.10 (-1.08 to 1.29, p = 0.863)	-0.59 (-2.35 to 1.16, p = 0.507)	-0.61 (-2.89 to 1.67, p = 0.600)
**Supplements**	0.80 (-0.65 to 2.25, p = 0.278)	0.33 (-1.21 to 1.88, p = 0.674)	-	0.44 (-1.04 to 1.92, p = 0.563)	-0.26 (-2.23 to 1.70, p = 0.794)	-0.28 (-2.72 to 2.17, p = 0.824)
**CBT**	0.36 (-0.68 to 1.41, p = 0.497)	-0.11 (-1.29 to 1.08, p = 0.861)	-0.44 (-1.91 to 1.04, p = 0.561)	-	-0.70 (-2.39 to 0.99, p = 0.418)	-0.72 (-2.95 to 1.52, p = 0.530)
**Tele-intervention**	1.07 (-0.61 to 2.75, p = 0.213)	0.60 (-1.17 to 2.37, p = 0.506)	0.27 (-1.70 to 2.24, p = 0.790)	0.70 (-1.00 to 2.41, p = 0.419)	-	-0.01 (-2.60 to 2.58, p = 0.994)
**Combination therapy**	1.09 (-1.17 to 3.34, p = 0.344)	0.62 (-1.69 to 2.94, p = 0.599)	0.29 (-2.19 to 2.76, p = 0.819)	0.72 (-1.55 to 3.00, p = 0.531)	0.03 (-2.59 to 2.64, p = 0.984)	-

Values given in SMD (95%CI); CBT, cognitive based therapy; SMD, standardised mean difference.

### Overall mortality

All treatment modalities did not affect the overall mortality as compared to standard care with moderate to high certainty of results following the GRADE score (Table 1). When compared to standard care, antidepressants (RR: 0.54, 95% CI: 0.13 to 2.29, p = 0.406), CBT (RR: 0.98, 95% CI: 0.80 to 1.19, p = 0.847), combination therapy (RR: 1.28, 95% CI: 0.70 to 2.34, p = 0.423) and psychosocial therapy (RR: 1.34, 95% CI: 0.83 to 2.16, p = 0.227) did not significantly affect the overall mortality risk. When comparing between the treatment modalities, there were no significant differences between all six treatment modalities in affecting the overall mortality (Table 3), and the heterogeneity within the comparisons were noted to be high (I^2^ = 89.4%). Psychosocial therapy did not decrease overall mortality when compared to antidepressants (RR: 2.27, 95% CI: 0.54 to 9.39, p = 0.263) or CBT (RR: 1.35, 95% CI: 0.81 to 2.23, p = 0.253). Treatment with antidepressants also did not reduce the overall mortality when compared to CBT (RR: 0.59, 95% CI: 0.15 to 2.32, p = 0.455).

**Table 3:** Summary of network analysis for overall mortality.

	Psychosocial therapy	Antidepressants	Supplements	CBT	Tele-intervention	Combination therapy
**Psychosocial therapy**	-	2.27 (0.54 to 9.39, p = 0.263)	1.32 (0.03 to 65.37, p = 0.890)	1.35 (0.81 to 2.23, p = 0.253)	1.20 (0.02 to 60.95, p = 0.930)	1.03 (0.48 to 2.20, p = 0.941)
**Antidepressants**	0.44 (0.11 to 1.84, p = 0.263)	-	0.58 (0.01 to 35.52, p = 0.797)	0.59 (0.15 to 2.32, p = 0.455)	0.53 (0.01 to 33.12, p = 0.762)	0.45 (0.10 to 1.99, p = 0.297)
**Supplements**	0.76 (0.02 to 38.09, p = 0.890)	1.72 (0.03 to 104.58, p = 0.797)	-	1.02 (0.02 to 49.90, p = 0.992)	0.90 (0.00 to 223.63, p = 0.972)	0.78 (0.02 to 39.65, p = 0.902)
**CBT**	0.74 (0.45 to 1.23, p = 0.253)	1.68 (0.43 to 6.55, p = 0.455)	0.98 (0.02 to 47.94, p = 0.992)	-	0.89 (0.02 to 44.70, p = 0.952)	0.76 (0.41 to 1.45, p = 0.411)
**Tele-intervention**	0.84 (0.02 to 43.38, p = 0.930)	1.90 (0.03 to 119.10, p = 0.762)	1.11 (0.00 to 273.14, p = 0.971)	1.13 (0.02 to 56.83, p = 0.952)	-	0.86 (0.02 to 45.15, p = 0.942)
**Combination therapy**	0.97 (0.45 to 2.08, p = 0.941)	2.20 (0.50 to 9.58, p = 0.297)	1.28 (0.03 to 64.72, p = 0.902)	1.31 (0.69 to 2.46, p = 0.411)	1.16 (0.02 to 60.34, p = 0.942)	-

Values given in RR (95%CI); CBT, cognitive based therapy; RR, Risk Ratio.

### Cardiac mortality

Compared to standard care, none of the treatment modalities was found to significantly reduce the cardiac mortality with moderate certainty of results following the GRADE score (Table 1). Further, each of these treatment modalities did not significantly differ from one another in their effects on cardiac mortality (S4 Table), and the heterogeneity within the comparisons was low (I^2^ = 0.0%). The cardiac mortality among patients who received psychosocial therapy was similar to those who received CBT (RR: 1.65, 95% CI: 0.93 to 2.92, p = 0.085), or antidepressants (RR: 1.48, 95% CI: 0.03 to 75.19, p = 0.846). Treatment with antidepressants or CBT was also associated with similar cardiac mortality (RR: 1.12, 95% CI: 0.02 to 55.70, p = 0.956).

### Myocardial infarction

MI was reported in studies that used psychosocial therapy, antidepressant, and CBT only. These 3 treatment modalities did not significantly affect the incidence of MI as compared to standard care, with moderate certainty of results following the GRADE (Table 1). Their effects on the incidence of MI also did not differ significantly from one another (S5 Table), and the heterogeneity within the comparisons was low (I^2^ = 0.0%). Patients who received psychosocial therapy experienced similar risk of subsequent MI in comparison to antidepressants (RR: 1.19, 95% CI: 0.44 to 3.19, p = 0.728), or CBT (RR: 1.01, 95% CI: 0.61 to 1.67, p = 0.978). Treatment with antidepressants or CBT was also found to have no difference in the risk of subsequent MI (RR: 0.84, 95% CI: 0.35 to 2.08, p = 0.713).

## Discussion

**Fig 4:**
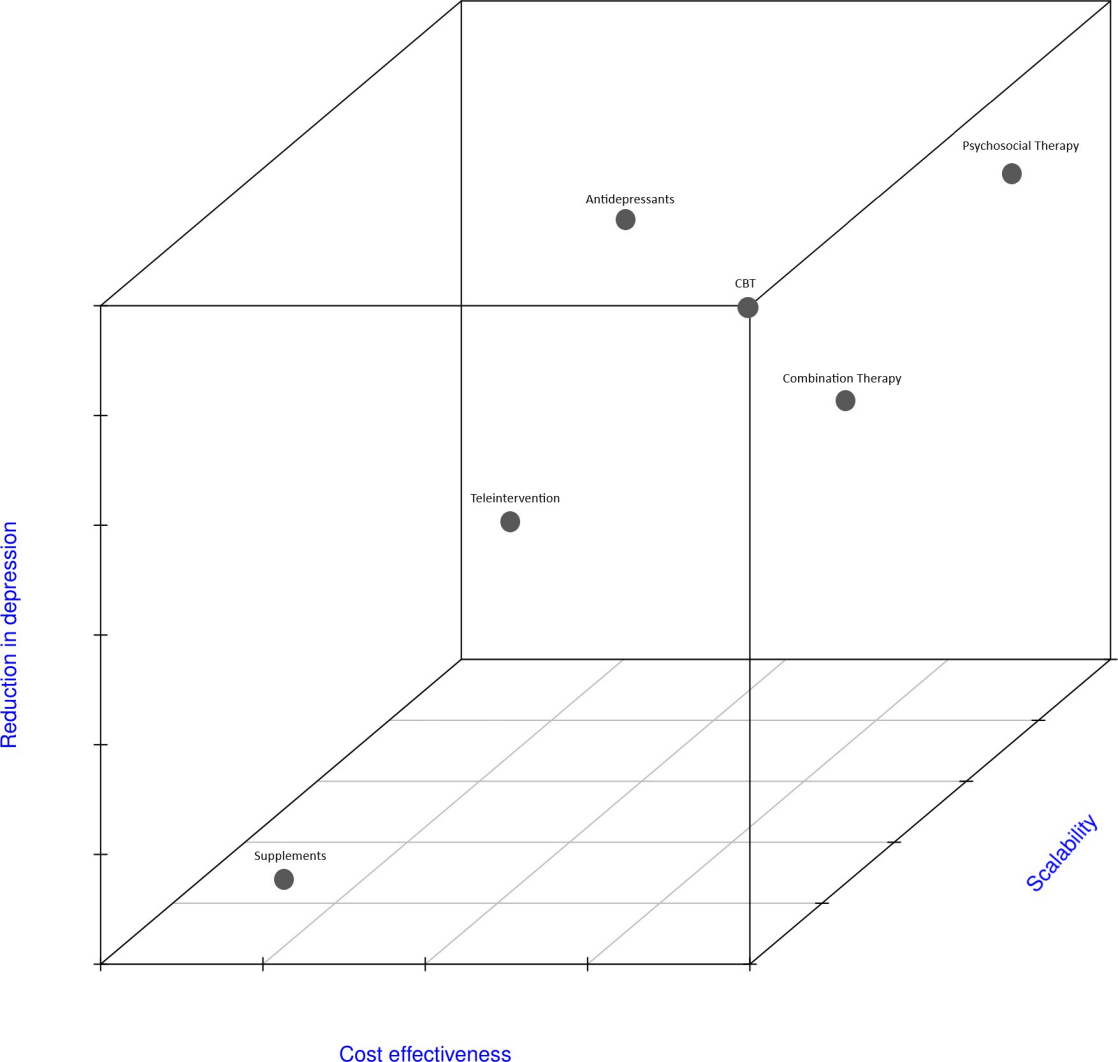
Effectiveness of interventions based on cost-effectiveness, reduction in depression and scalability.

This network meta-analysis is the first to examine the effects of various psychological interventions in the treatment of depression after ACS (Fig 4). Statements from the American Heart Association (AHA) and the European Society of Cardiology (ESC) both identified post ACS depression to be a significant risk factor for cardiac morbidity and mortality [63,64], with recent consensus describing the importance of regular screening for depression in patients suffering from coronary artery disease with consideration of CBT and SSRIs for the treatment of their depression [65]. While previous meta-analysis has examined the effects of CBT [66] and the use of SSRIs [67,68] in treating depression after ACS, no study has done a ranking assessment between all the possible treatments for post-ACS depression. Thus, we add to these findings by (1) conducting a network meta-analysis between treatments (2) pooling the effects of intervention on clinical outcomes. The main findings of the present study include the identification of a large reduction in depression scores upon initiation of any psychological interventions aside from the use of non-pharmacological supplements. However, there was no significant association in psychological interventions with prognostic outcomes such as overall mortality, cardiac death, or MI. Interestingly, there were no statistically significant differences between different interventions in the reduction of depression scores.

The largest reduction in depression scores was observed with psychosocial therapy (SMD: -1.21), CBT (SMD: -0.75), and antidepressant use (SMD: -0.73). Though psychosocial therapy and CBT is shown to be more effective in the reduction of depression scores compared to usual care with antidepressants, its effectiveness is dependent on many factors, such as the qualification of the healthcare professional in charge and the receptivity of the patient to treatment, which can also limit its scalability [69]. Additionally, both are also restricted by the fact that the execution of these specialized treatment may be limited from a lack of sufficiently trained mental health professionals to meet the growing demands [70,71]. In contrast, though antidepressants has been noted to be less effective than CBT and psychosocial therapy, they offer a cost effective and scalable alternative for areas without sufficiently trained mental health professionals. However, there exists limitations to the use of antidepressants. Antidepressants provide symptomatic relief but do not address the root cause of post-ACS depression. They also require prolonged usage to prevent relapse, which is not without its adverse effects [72]. Moreover, most-anti-depressants are associated with prolonged QT, which can predispose patients to arrhythmic events, which can worsen their outcomes post-ACS. This may place additional burden on the healthcare system with closer surveillance of its associated adverse effects, that may lead to further investigations and treatment. Although SSRIs are the first line treatment for depression [73], they are cytochrome P450 inhibitors which can potentially alter the pharmacokinetics of statins commonly prescribed after ACS [74]. Additionally, up to 60% of ACS patients are elderly who are commonly subjected to polypharmacy due to the presence of multiple comorbidities [75,76].

In this network analysis, reduction in depression score and prognostic outcomes associated with tele-intervention was not statistically different from those of antidepressants, CBT or psychosocial therapy. Tele-intervention has been widely used in the management of chronic conditions other than post-ACS depression with promising results and these include stroke [77], chronic obstructive pulmonary disease [78]. The role of tele-intervention encompasses monitoring for symptoms and quality of life, as well as providing timely treatment remotely in patients afflicted with coronary artery disease [79]. Tele-intervention has, in recent times, been widely implemented during the present SARS-CoV-2 pandemic that limits accessibility to consultation and treatment with encouraging outcomes for both patients with mental health conditions [80] or ACS [81–83]. Additionally, tele-intervention provide a wider outreach to rural societies that can often be neglected by healthcare systems [84]. Alternative treatments such as electroconvulsive therapy is a rapidly evolving treatment modality for depression which has proven to be highly effective [85]. However, more studies have to be conducted on its safety and efficacy in post-ACS patients [86].

Despite reduction in depression scores, current analysis did not find any interventions that significantly reduce overall mortality, cardiac death, or MI. In a meta-analysis of 6367 patients, van Melle et al found that suffering from depression after ACS is associated with 2 to 2.5 times increased risk of death and MI [9]. However, beyond the effects on clinical outcomes, treating depression can result in an improvement in quality of life. Improving the dignity of patients has since been a key metric of the healthcare system performance. Additionally, while the treatment of depression may not directly affect clinical outcomes, patients with depression are often less adherent to treatment which, in turn, can affect the adherence of medications used after ACS treatment, leading to an indirect adverse impact on clinical outcomes [10,11,87].

### Limitations

The present analysis represents the largest network analysis of randomized controlled trials for the treatment of depression after ACS. However, there are a few limitations. The studies included in current analysis were predominantly conducted in the West with approximately 10% of the studies conducted in the East, and it remains to be seen if the results are transferable. Moreover, the analysis of clinical outcomes was not adjusted for established other cardiac prognostic factors (such as left ventricular ejection fraction, kidney impairment etc) as the data were not available. Furthermore, we were unable to control for the varied scales used to measure depression scores, due to insufficient included articles examining each outcome, which greatly affected the impression of the severity of depression of the patients examined and the accuracy of the change in their scores. To help reduce the effect of the latter on our analysis, we opted to use SMD, which helps reduce the variability in the measurement different scores. However, some caution should be done in interpreting our results on the improvement of depression scores as it would not completely remove all differences and biases between the various depression scales used in the included papers. Additionally, all of our analyses derived from the network meta-analyses were indirect comparisons, and there was a lack of direct head-to-head comparisons between the six different forms of treatment. All of the included studies compared their intervention to usual care, which may have added additional biases and imprecision to our study. Future head-to-head studies comparing the different interventions should be considered, which can improve future research and aid future treatment decisions. Next, there was large variations in the duration of follow-up times between each study, and thus some long-term outcomes, such as cardiac mortality and MI, may not be truly reflective of the clinical outcome. Additionally, we were unable to incorporate a cost-effective analysis of various treatment modalities used in the studies included in this analysis.

## Conclusions

This network meta-analysis found that psychological interventions, including psychosocial therapy, antidepressants, CBT, tele-intervention and/or combination therapy, have an important role in alleviating symptoms of depression in patients who have suffered from ACS. No significant difference in treatment effect of the various psychological treatment modalities on depression. Future longer duration studies on psychological interventions, and head-to-head studies assessing the clinical outcomes and adherence to ACS-specific interventions will be needed, which can help to improve the precision of the future analyses on this important topic.

## Supporting information

S1 Table Full search strategy. (DOCX)

S2 Table Cochrane risk-of-bias 2 tool for included articles. (DOCX)

S3 Table Summary of included articles. (DOCX)

S4 Table Summary of network analysis for cardiac mortality. (DOCX)

S5 Table Summary of network analysis for myocardial infarction. (DOCX)

S6 Table PRISMA checklist of items to include when reporting a systematic review involving a network meta-analysis. (DOCX)

S1 Appendix Funnel plots. (DOCX)

S2 Appendix Summary of direct and indirect evidence for the various network meta-analysis outcomes. (DOCX)
