# Supplementary material for: A network meta-analysis of 12,116 individuals from randomized controlled trials in the treatment of depression after acute coronary syndrome
Source: PLoS One. 2022 Nov 30;17(11):e0278326. doi: 10.1371/journal.pone.0278326 (PMC9710843; doi:10.1371/journal.pone.0278326)
Supplement: S1 Table — (DOCX) [file pone.0278326.s001.docx]

**S1 Table: Full Search Strategy for Medline**

| S/N | Search Strategy |
| --- | --- |
| 1 | exp psychotherapy/ or counseling.tw. or exp directive counseling/ or exp distance counseling/ or stress management.tw. or exp cardiac rehabilitation/ |
| 2 | exp depression/ or depress*.tw. |
| 3 | exp acute coronary syndrome/ or exp angina, unstable/ or exp myocardial infarction/ or ST elevat* myocardial infarction.tw. or non-ST elevat* myocardial infarction.tw. or unstable angina.tw. or acute angina.tw. |
| 4 | 1 or 2 |
| 5 | 3 and 4 |
| 6 | limit 5 to English |
| 7 | exp animals/ not humans.sh. |
| 8 | 6 not 7 |
| 9 | (randomized controlled trial or controlled clinical trial).pt. or randomized.ab. or placebo.ab. or clinical trials as topic.sh. or randomly.ab. or trial.ti. or randomised controlled trial.sh. |
| 10 | 8 and 9 |
