# Supplementary material for: A network meta-analysis of 12,116 individuals from randomized controlled trials in the treatment of depression after acute coronary syndrome
Source: PLoS One. 2022 Nov 30;17(11):e0278326. doi: 10.1371/journal.pone.0278326 (PMC9710843; doi:10.1371/journal.pone.0278326)
Supplement: S2 Table — (DOCX) [file pone.0278326.s002.docx]

Table **S2 Table:** Cochrane Risk-of-Bias 2 tool for Included Articles

|  | **D1** | **D2** | **D3** | **D4** | **D5** | **Overall** |
| --- | --- | --- | --- | --- | --- | --- |
| Bagherian et al 2016 | 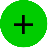 | 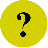 | 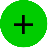 | 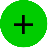 | 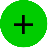 | 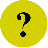 |
| Berkman et al 2003 | 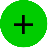 | 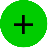 | 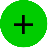 | 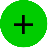 | 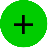 | 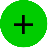 |
| Davidson et al 2013 | 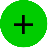 | 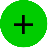 | 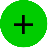 | 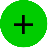 | 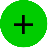 | 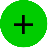 |
| Davidson et al 2010 | 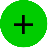 | 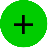 | 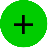 | 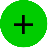 | 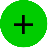 | 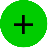 |
| Fernandes et al 2017 | 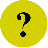 | 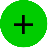 | 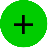 | 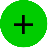 | 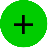 | 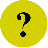 |
| Follick et al 1988 | 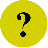 | 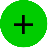 | 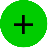 | 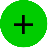 | 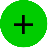 | 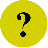 |
| Frasure-Smith DOI 1997 | 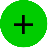 | 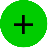 | 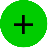 | 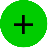 | 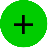 | 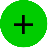 |
| Ghiasi et al 2018 | 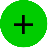 | 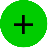 | 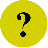 | 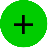 | 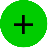 | 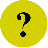 |
| Giltay et al 2011 | 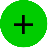 | 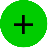 | 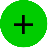 | 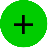 | 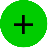 | 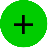 |
| Glassman et al 2009 | 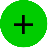 | 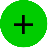 | 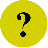 | 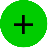 | 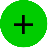 | 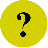 |
| Haberka et al 2013 | 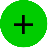 | 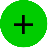 | 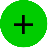 | 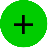 | 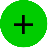 | 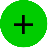 |
| Honig et al 2007 | 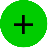 | 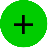 | 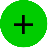 | 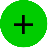 | 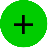 | 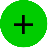 |
| Humphries et al 2021 | 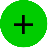 | 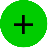 | 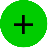 | 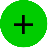 | 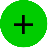 | 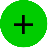 |
| Jørstad et al 2016 | 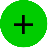 | 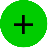 | 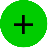 | 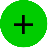 | 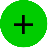 | 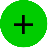 |
| Kim et al 2015 | 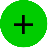 | 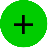 | 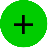 | 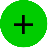 | 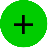 | 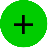 |
| Kronish et al 2020 | 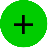 | 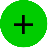 | 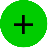 | 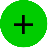 | 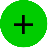 | 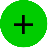 |
| Liang et al 2019 | 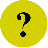 | 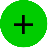 | 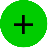 | 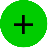 |  |  |
| McLaughlin et al 2005 |  |  |  |  |  |  |
| Mohapatra et al 2005 |  |  |  |  |  |  |
| Moludi et al 2019 |  |  |  |  |  |  |
| O'Neil et al 2014 |  |  |  |  |  |  |
| O'Neil et al 2015 |  |  |  |  |  |  |
| Rafanelli et al 2020 |  |  |  |  |  |  |
| Roncella et al 2013 |  |  |  |  |  |  |
| Schneider et al 2020 |  |  |  |  |  |  |
| Stern et al 1983 |  |  |  |  |  |  |
| Strik et al 2000 |  |  |  |  |  |  |
| Turner et al 2014 |  |  |  |  |  |  |
| Wang et al 2016 |  |  |  |  |  |  |
| Warber et al 2011 |  |  |  |  |  |  |

**Domains:**

D1: Bias arising from the randomization process

D2: Bias due to deviations from intended interventions

D3: Bias due to missing outcome data

D4: Bias in measurement of the outcome

D5: Bias in selection of reported results

|  | Low risk of bias |
| --- | --- |
|  | High risk of bias |
|  | Unclear risk of bias |
