# Supplementary material for: A network meta-analysis of 12,116 individuals from randomized controlled trials in the treatment of depression after acute coronary syndrome
Source: PLoS One. 2022 Nov 30;17(11):e0278326. doi: 10.1371/journal.pone.0278326 (PMC9710843; doi:10.1371/journal.pone.0278326)
Supplement: S3 Table — (DOCX) [file pone.0278326.s003.docx]

**S3 Table:** Summary of Included Articles

| **Author (Year)** | **Country** | **Intervention** | **Sample Size** | **Length of follow-up (months)** | **Mean Age** | | **Gender (female)** | | **Depression Scale** | **Depression score (Baseline)** | |
| --- | --- | --- | --- | --- | --- | --- | --- | --- | --- | --- | --- |
|  |  |  |  |  | **Intervention** | **Usual Care** | **Intervention** | **Usual Care** |  | **Intervention** | **Usual Care** |
| Stern et al  (1983) | US | Psychosocial therapy | 64 | 3 | - | - | 0.114 | 0.759 | Zung Self-Rating Depression Scale | 33.71 | 37.44 |
| Follick et al  (1988) | US | Antidepressant | 211 | 29 | 54.2 | 54.6 | 0.316 | 0.761 | SCL-90 | 0.71 | 0.71 |
| Frasure-Smith et al (1997) | Canada | Psychosocial therapy | 1376 | 6 | 59.3 | 59.3 | 0.338 | 0.651 | BDI | 8.1 | 8.4 |
| Strik et al  (2000) | Netherlands | Antidepressant | 54 | 9 | 54.1 | 58.7 | 0.222 | 0.63 | HAM-D | 22 | 21.2 |
| Berkman et al  (2003) | US | CBT | 2481 | 2 | 61 | 61 | 0.43 | 0.56 | BDI | 15.7 | 15.7 |
| McLaughlin et al  (2005) | US | Psychosocial therapy | 79 | 9 | 59.9 | 60.7 | 0.311 | 0.647 | HADS-D | 8.1 | 6.5 |
| Mohapatra et al  (2005) | India | Antidepressant | 17 | 12 | 55.2 | 56.8 | 0.545 | 0.667 | HAM-D | 15 | 16.67 |
| Honig et al  (2007) | Netherlands | Antidepressant | 91 | 2.75 | 56.6 | 57.9 | 0.128 | 0.818 | HAM-D | 18.66 | 16.81 |
| Glassman et al  (2009) | US | Antidepressant | 369 | 40 | 56.8 | 57.6 | 0.37 | 0.64 | HAM-D | 19.6 | 19.6 |
| Davidson et al (2010) | US | CBT | 157 | 6 | 59.3 | 61.1 | 0.54 | 0.47 | BDI | 19 | 19.6 |
| Giltay et al  (2011) | US | Supplements | 4116 | 1 | 68.8 | 68.7 | 0.21 | 0.8 | GDS-15 | 1.337 | 1.36 |
| Warber et al  (2011) | US | Psychosocial therapy | 47 | 27 | 60.486 | 61 | 0.46 | 0.8 | BDI | 11.622 | 8 |
| Davidson et al  (2013) | US | CBT | 150 | 12 | 59.2 | 60 | 0.411 | 0.571 | BDI | 21 | 20.6 |
| Haberka et al  (2013) | Poland | Supplements | 52 | 12 | 56.4 | 59.6 | 0.13 | 0.82 | BDI | 11.1 | 11.7 |
| Roncella et al  (2013) | Italy | Psychosocial therapy | 94 | 12 | 55 | 55 | 0.089 | 0.867 | BDI | 5.25 | 6.75 |
| Turner et al  (2014) | Australia | CBT | 42 | 18 | 55.6 | 57 | 0.143 | 0.714 | BDI | 20.32 | 19.99 |
| O'Neil et al  (2014) | Australia | Tele-intervention | 297 | 0.25 | 62 | 59.7 | 0.213 | 0.795 | HADS-D | 4.5 | 4.3 |
| Kim et al  (2015) | South Korea | Antidepressant | 300 | 6 | 60.164 | 59.3 | 0.417 | 0.732 | HAM-D | 15.88 | 14.9 |
| O'Neil et al  (2015) | Australia | Tele-intervention | 121 | 6 | 61 | 58.9 | 0.262 | 0.767 | PHQ-9 | 9 | 9.4 |
| Bagherian et al  (2016) | Iran | Psychosocial therapy | 48 | 3 | 54.8 | 49.9 | 0.292 | 0.792 | HADS-D | 15.7 | 15.2 |
| Jørstad et al  (2016) | Netherlands | Psychosocial therapy | 120 | 6 | 57.3 | 58.1 | 0.22 | 0.77 | BDI | 8.1 | 6.1 |
| Wang et al  (2016) | Singapore | Psychosocial therapy | 128 | 12 | 54.9 | 55.8 | 0.094 | 0.891 | HADS-D | 3.02 | 3.59 |
| Fernandes et al  (2017) | Portugal | Psychosocial therapy | 121 | 30 | 61.77 | 66.11 | 0.313 | 0.714 | HADS-D | 12.8 | 9.61 |
| Ghiasi et al  (2018) | Iran | CBT | 30 | 12 | 56.6 | 56.6 | 0.4 | 0.467 | GHQ-28 | 37.87 | 42.6 |
| Liang et al  (2019) | China | Psychosocial therapy | 116 | 1 | 55.12 | 55.41 | 0.431 | 0.551 | SDS | 49.16 | 49.29 |
| Moludi et al  (2019) | Iran | Supplements | 44 | 12 | 56.7 | 57.1 | 0.1 | 0.95 | BDI | 20.1 | 17.61 |
| Rafanelli et al  (2020) | Italy | CBT | 100 | 6.25 | 57.64 | 60.02 | 0.38 | 0.76 | CID | 7.92 | 6.9 |
| Kronish et al  (2020) | US | Combination therapy | 999 | 12 | 66.2 | 65.8 | 0.285 | 0.71 | CESD-10 | 4.91 | 4.74 |
| Schneider et al  (2020) | Canada | CBT | 53 | 4 | 56.72 | 59.29 | 0.48 | 0.321 | PHQ-9 | 11 | 9.18 |
| Humphries et al  (2021) | Sweden | CBT | 239 | 6 | 58.4 | 60.8 | 0.376 | 0.705 | HADS-D | 9.9 | 10.3 |

Legend: BDI, Beck Depression Inventory; CBT, cognitive based therapy; CESD-10, Center for Epidemiologic Studies Depression scale; CID, Clinical Interview for Depression; GDS-15, Geriatric Depression Scale; GHQ-28, General Health Questionnaire; HADS-D, Depression subscale of the Hospital Anxiety and Depression Scale; HAM-D, Hamilton Depression Rating Scale; PHQ-9, Patient Health Questionnaire 9; SCL-90, Symptom Checklist; SDS, Self-Rating Depression Scale
