# Supplementary material for: A network meta-analysis of 12,116 individuals from randomized controlled trials in the treatment of depression after acute coronary syndrome
Source: PLoS One. 2022 Nov 30;17(11):e0278326. doi: 10.1371/journal.pone.0278326 (PMC9710843; doi:10.1371/journal.pone.0278326)
Supplement: S4 Table — (DOCX) [file pone.0278326.s004.docx]

**S4 Table:** Summary of Network Analysis for Cardiac Mortality

|  | **Psychosocial therapy** | **Antidepressants** | **Supplements** | **CBT** | **Tele-intervention** |
| --- | --- | --- | --- | --- | --- |
| **Psychosocial therapy** | - | 1.48 (0.03 to 75.19, p=0.846) | 1.39 (0.03 to 69.41, p=0.871) | 1.65 (0.93 to 2.92, p=0.085) | 1.26 (0.02 to 64.72, p=0.911) |
| **Antidepressants** | 0.68 (0.01 to 34.47, p=0.846) | - | 0.94 (0.00 to 230.44, p=0.982) | 1.12 (0.02 to 55.70, p=0.956 | 0.85 (0.00 to 212.72, p=0.953) |
| **Supplements** | 0.72 (0.01 to 36.23, p=0.871) | 1.06 (0.00 to 262.43, p=0.982) | - | 1.19 (0.02 to 58.56, p=0.930) | 0.90 (0.00 to 223.63, p=0.972) |
| **CBT** | 0.61 (0.34 to 1.07, p=0.085) | 0.90 (0.02 to 44.70, p=0.956) | 0.84 (0.02 to 41.26, p=0.930) | - | 0.76 (0.01 to 38.09, p=0.891) |
| **Tele-intervention** | 0.79 (0.02 to 41.26, p=0.911) | 1.17 (0.00 to 295.89, p=0.953) | 1.11 (0.00 to 273.14, p=0.972) | 1.32(0.03 to 66.69, p=0.891) | - |

Values given in RR (95%CI); CBT, cognitive based therapy; RR, Risk Ratio
