# Supplementary material for: A network meta-analysis of 12,116 individuals from randomized controlled trials in the treatment of depression after acute coronary syndrome
Source: PLoS One. 2022 Nov 30;17(11):e0278326. doi: 10.1371/journal.pone.0278326 (PMC9710843; doi:10.1371/journal.pone.0278326)
Supplement: S5 Table — (DOCX) [file pone.0278326.s005.docx]

**S5 Table:** Summary of Network Analysis for Myocardial Infarction

|  | **Psychosocial therapy** | **Antidepressants** | **CBT** |
| --- | --- | --- | --- |
| **Psychosocial therapy** | - | 1.19 (0.44 to 3.19, p=0.728) | 1.01 (0.61 to 1.67, p=0.978) |
| **Antidepressants** | 0.84 (0.31 to 2.25, p=0.728) | - | 0.84 (0.35 to 2.08, p=0.713) |
| **CBT** | 0.99 (0.60 to 1.63, p=0.978) | 1.19 (0.48 to 2.89, p=0.713) | - |

Values given in RR (95%CI); CBT, cognitive based therapy; RR, Risk Ratio
