# Supplementary material for: A network meta-analysis of 12,116 individuals from randomized controlled trials in the treatment of depression after acute coronary syndrome
Source: PLoS One. 2022 Nov 30;17(11):e0278326. doi: 10.1371/journal.pone.0278326 (PMC9710843; doi:10.1371/journal.pone.0278326)
Supplement: S1 Appendix — (DOCX) [file pone.0278326.s007.docx]

**S1 Appendix:** Funnel Plots

*Supplementary Figure 1: Funnel Plot of Depression Score Analysis*


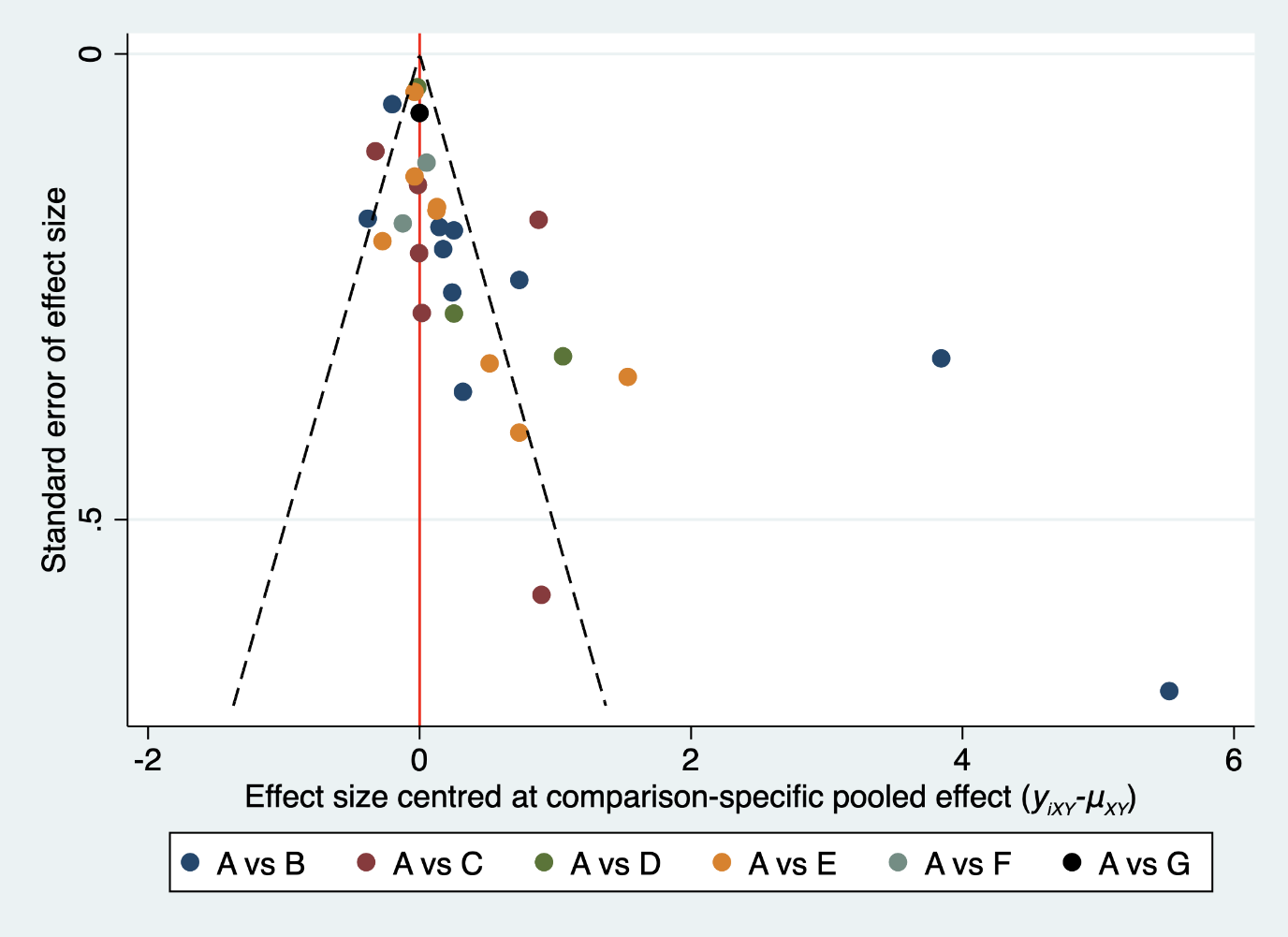


Legend: A, Standard Care; B, Psychosocial therapy; C, Anti-depressants; D, Supplements; E, Cognitive Behavorial Therapy; F, Tele-intervention; G, Anti-depressants with Cognitive behavorial therapy

*Supplementary Figure 2: Funnel Plot of Overall Mortality*


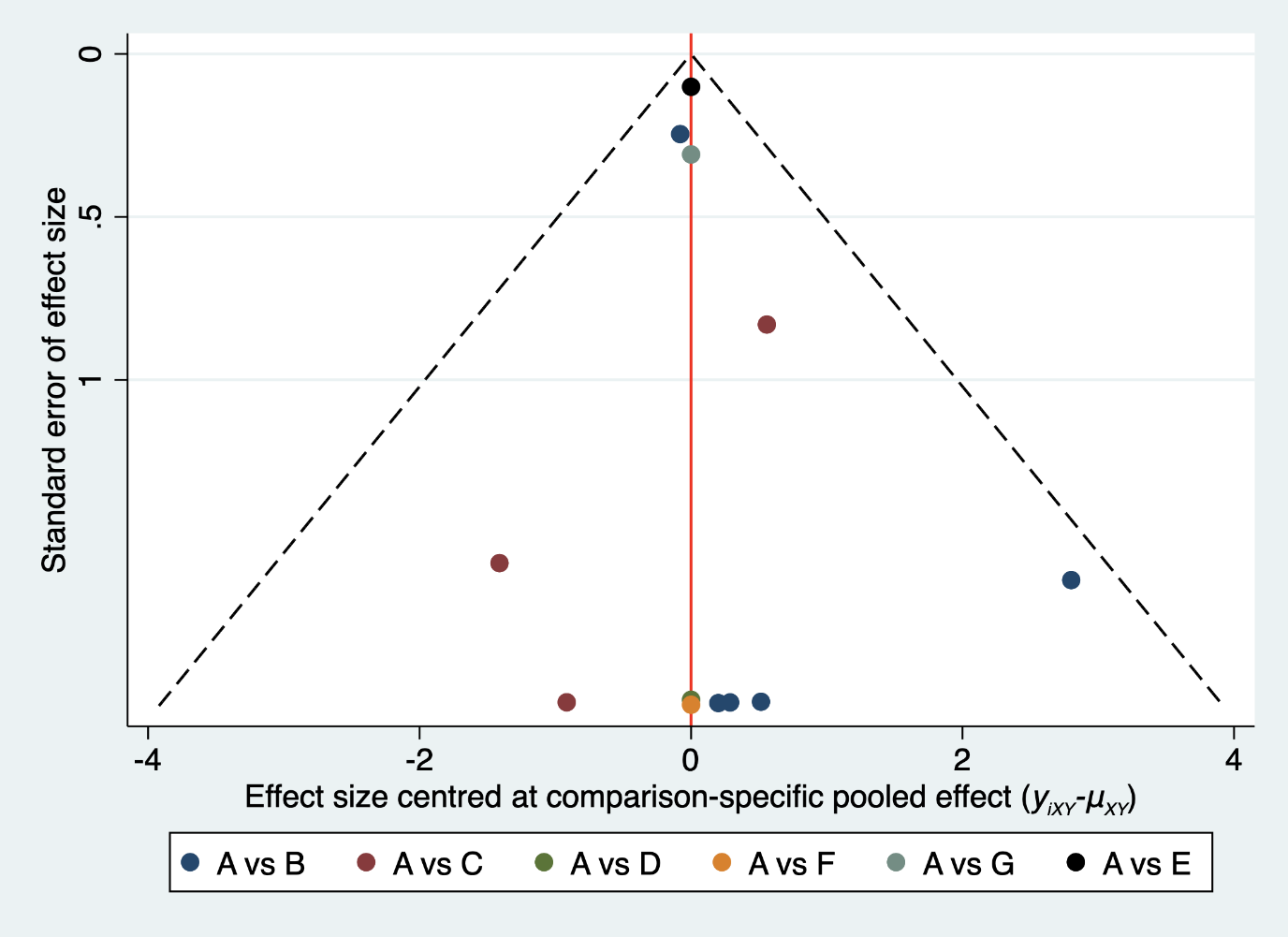


Legend: A, Standard Care; B, Psychosocial therapy; C, Anti-depressants; D, Supplements; E, Cognitive Behavorial Therapy; F, Tele-intervention; G, Anti-depressants with Cognitive behavorial therapy

*Supplementary Figure 3: Funnel Plot of Cardiac Mortality
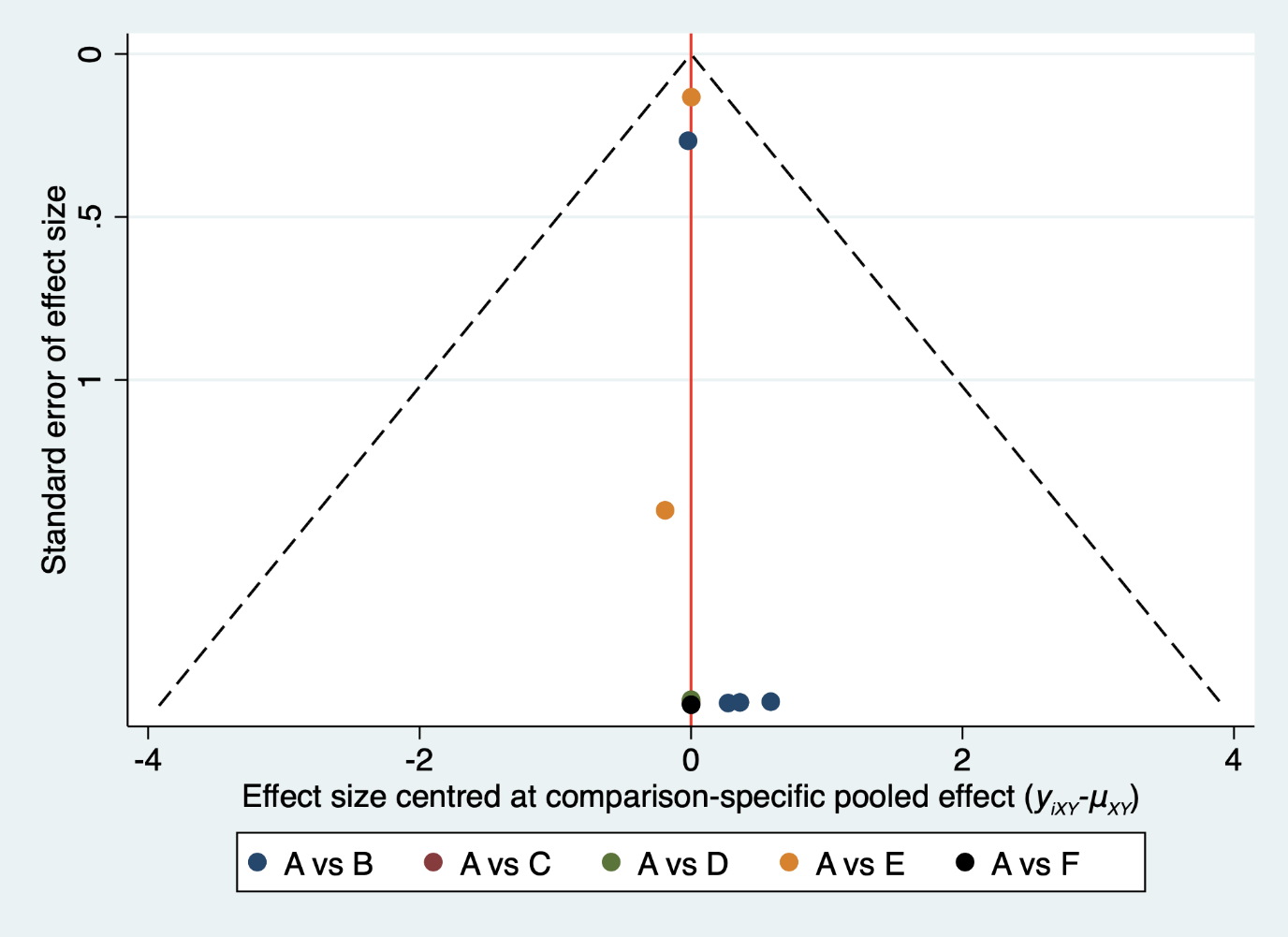
*

Legend: A, Standard Care; B, Psychosocial therapy; C, Anti-depressants; D, Supplements; E, Cognitive Behavorial Therapy; F, Tele-intervention; G, Anti-depressants with Cognitive behavorial therapy
