# Supplementary material for: A network meta-analysis of 12,116 individuals from randomized controlled trials in the treatment of depression after acute coronary syndrome
Source: PLoS One. 2022 Nov 30;17(11):e0278326. doi: 10.1371/journal.pone.0278326 (PMC9710843; doi:10.1371/journal.pone.0278326)

**S2 Appendix:** Summary of Direct and Indirect evidence for the various Network Meta-analysis outcomes

*Supplementary Figure 1:* *Visual Plot Showing the Proportions of Direct and Indirect Evidence in The Depression Score Comparison*


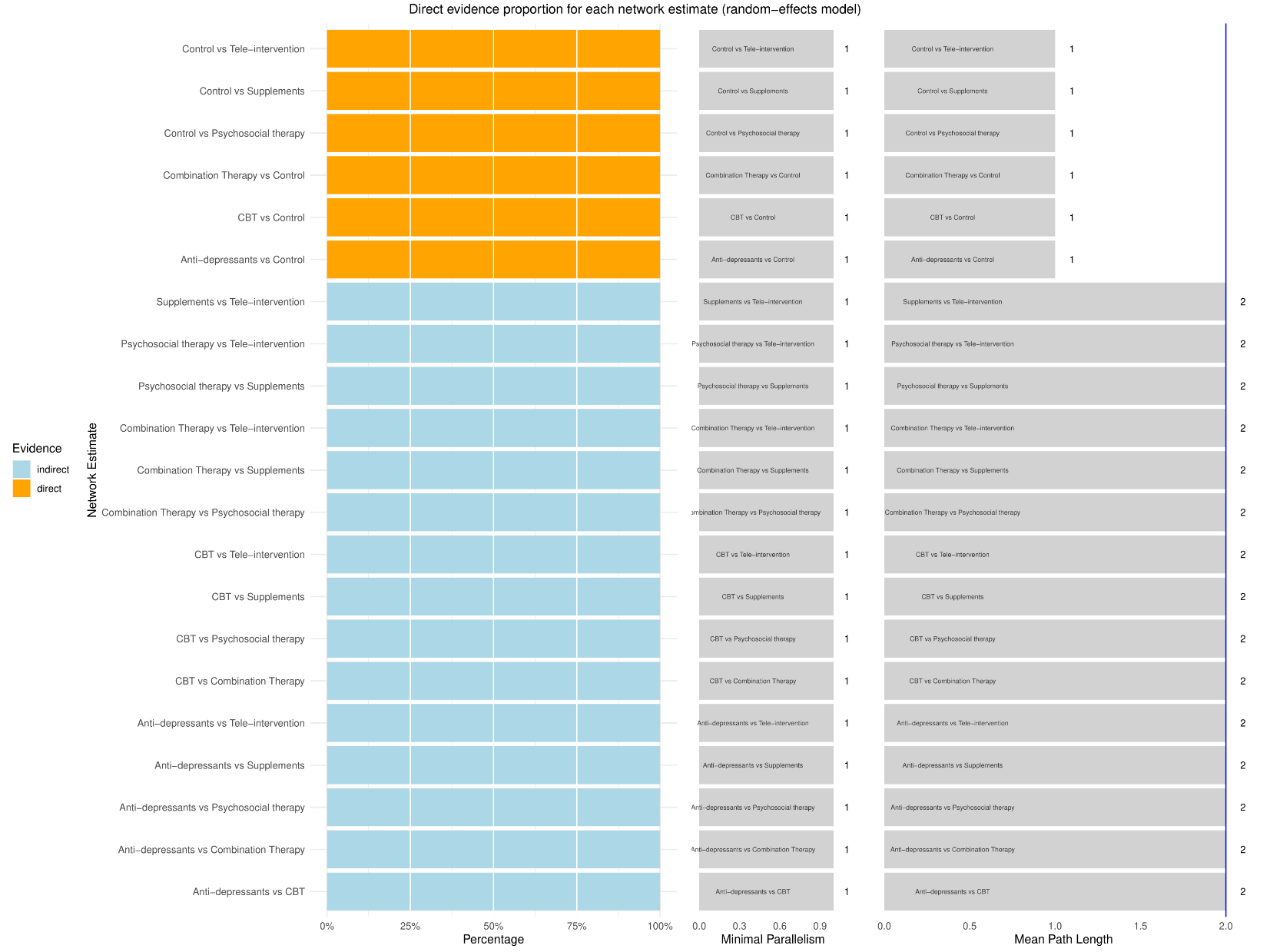


*Supplementary Figure 2: Visual Plot Showing the Proportions of Direct and Indirect Evidence in The Overall Mortality Comparison*


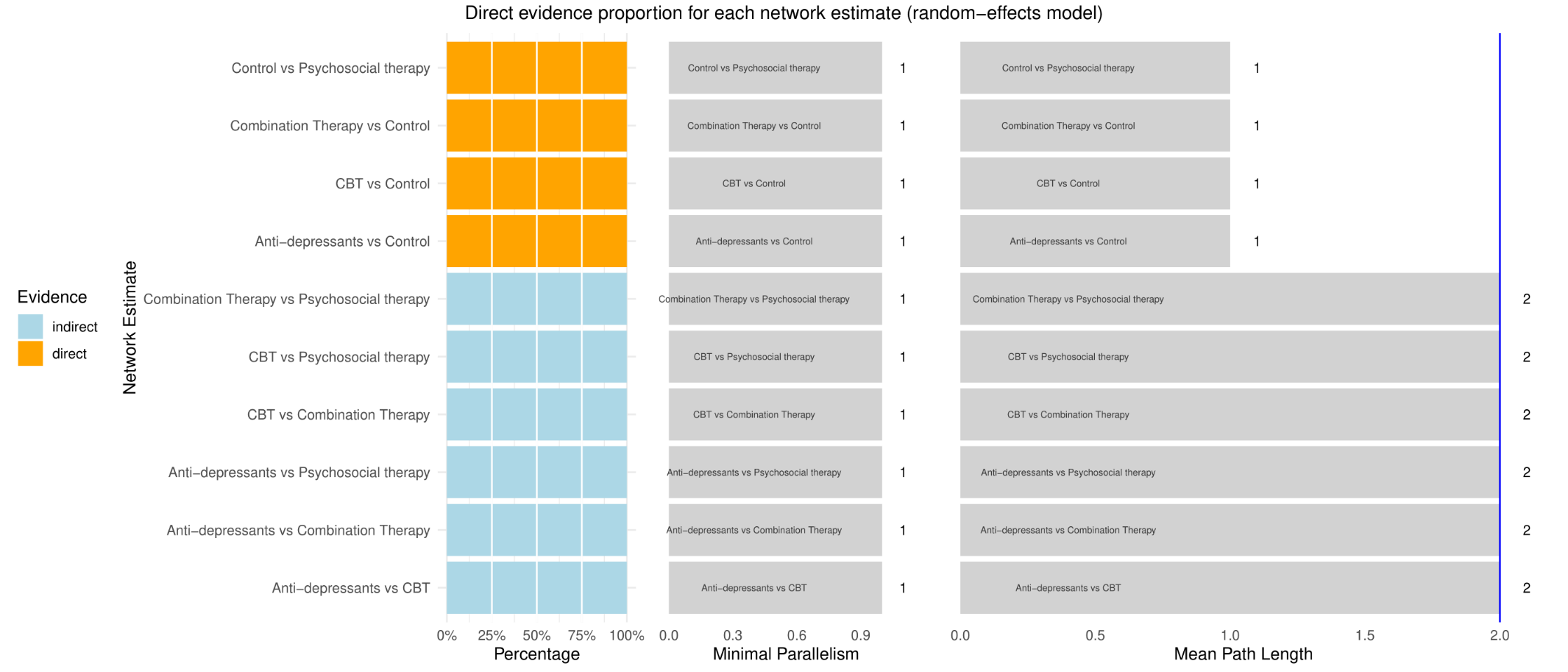


*Supplementary Figure 3: Visual Plot Showing the Proportions of Direct and Indirect Evidence in The Cardiac Mortality Comparison*


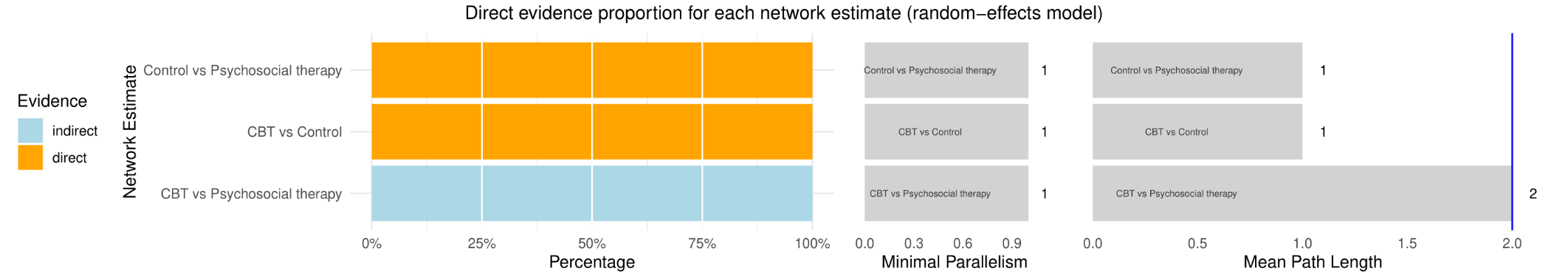


*Supplementary Figure 4: Visual Plot Showing the Proportions of Direct and Indirect Evidence in the Myocardial Infarction Comparison*


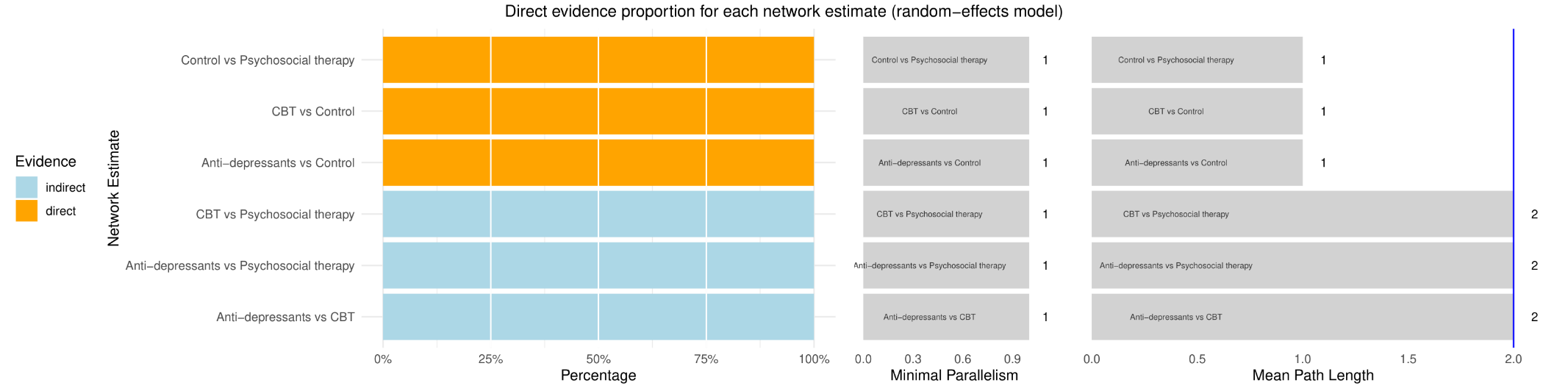

Supplement: S2 Appendix — (DOCX) [file pone.0278326.s008.docx]
